# Chirality Effects and Semiconductor versus Metallic Nature in Halide Nanotubes

**DOI:** 10.1021/acs.jpcc.3c00244

**Published:** 2023-04-05

**Authors:** Costanza Borghesi, Giacomo Tanzi Marlotti, Enric Canadell, Giacomo Giorgi, Riccardo Rurali

**Affiliations:** † Department of Civil & Environmental Engineering (DICA), Università degli Studi di Perugia, Via G. Duranti 93, 06125 Perugia, Italy; ‡ Department of Physics “Aldo Pontremoli”, Università degli Studi di Milano, Via Celoria 16, I-20133 Milano, Italy; § Institut de Ciència de Materials de Barcelona, ICMAB−CSIC, Campus UAB, 08193 Bellaterra, Spain; ∥ CIRIAF - Interuniversity Research Centre, University of Perugia, Via G. Duranti 93, 06125 Perugia, Italy; ⊥ CNR-SCITEC, 06123 Perugia, Italy

## Abstract

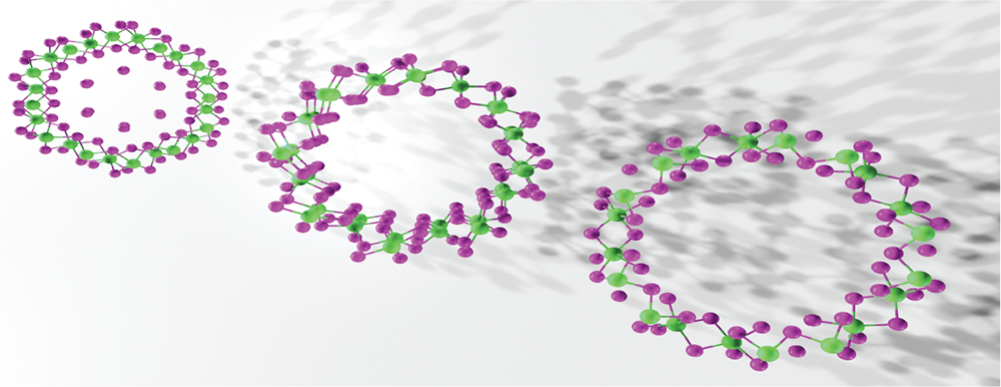

A density functional theory study of the electronic structure of nanostructures based on the hexagonal layers of LuI_3_ is reported. Both bulk and slabs with one to three layers exhibit large and indirect bandgaps. Different families of nanotubes can be generated from these layers. Semiconducting nanotubes of two different chiralities have been studied. The direct or indirect nature of the optical gaps depends on the chirality, and a simple rationalization of this observation based on band folding arguments is provided. Remarkably, a metastable form of the armchair LuI_3_ nanotubes can be obtained under a structural rearrangement such that some iodine atoms are segregated toward the center of the nanotube forming chains of dimerized iodines. These nanotubes having an Lu_2*N*_I_5*N*_ backbone are predicted to be metallic and should be immune toward a Peierls distortion. The iodine chains in the inner part of the nanotubes are weakly bound to the backbone so that it should be possible to remove these chains to generate a new series of neutral Lu_2*N*_I_5*N*_ nanotubes which could exhibit interesting magnetic behavior. Because the LuI_3_ structure occurs for a large number of lanthanide and actinide trihalides, a tuning of the optical, transport, and probably magnetic properties of these new families of nanotubes can be a challenging prospect for future experimental studies.

## Introduction

Since the isolation of monolayer graphene in 2004,^1^ the research in two-dimensional (2D) materials^2^ has become one of the most active fields in condensed matter and nanoscience. While at first most efforts were directed at overcoming some of graphene limitations, such as the absence of an electronic bandgap,^3^ the discovery of many other families of 2D materials^4−6^ has considerably broadened the scope of the research in this field.^7−10^ Graphene is still a privileged platform to explore the fundamental properties of 2D materials, but at the same time, novel properties that are unique of given classes of 2D materials are continuously emerging,^11^ such as in the recently discovered 2D magnetic materials.^12^

Importantly, even when the chemical composition is defined, in many cases the properties can still be tuned to a large extent by controlling the number of layers, the polymorph (when polymorphism exists), and the stacking order. A tight control on these features allows designing materials that are metals or semiconductors,^13,14^ that are direct or indirect bandgap semiconductors,^15^ and that have different selection rules of their vibrational modes.^16^ In this scenario, the recent capabilities of synthesizing van der Waals heterostructures,^17^ where single layers of different materials can be combined, or magic angle engineering,^18^ where the properties of a two-layer system can be tuned by controlling the misfit angle between them, make the capabilities of designing few-layer materials with tailor-made properties almost endless. Last, but not least, monolayers can be wrapped up to form nanotubes,^19−21^ where additional control parameters of the material properties are the diameter and the chirality, i.e., the axis around which the monolayer is rolled up. A carbon nanotube with (*n*, *m*) chiral indexes, for instance, is metallic when *n* – *m* = 3*l* with *l* an integer, while otherwise is a small bandgap semiconductor.^22^ Many inorganic tubular nanostructures, other than carbon, have been reported to date,^23^ mostly based on boron nitride, transition-metal dichalcogenides, and misfit layered compounds.^24^ The formation of single-walled NTs is in general challenging because the required compensation of the large bending energy involved tends to favor the formation of multiwall NTs.^23^ To bypass this limitation, the use of carbon NTs or other stable tubular nanostructures as growth templates has been successfully reported in several systems.^21,25,26^

For all the aforementioned reasons, the discovery and the synthesis of a new family of 2D materials have multiple implications. On the one hand, these new materials can have novel properties by themselves that might be of interest for specific applications; on the other hand, they can give rise to new van der Waals heterostructures in combination with single layers of other materials. Finally, a tubular one-dimensional (1D) nanostructure, whose properties depend on diameter and chirality, might exist.

**Figure 1:**
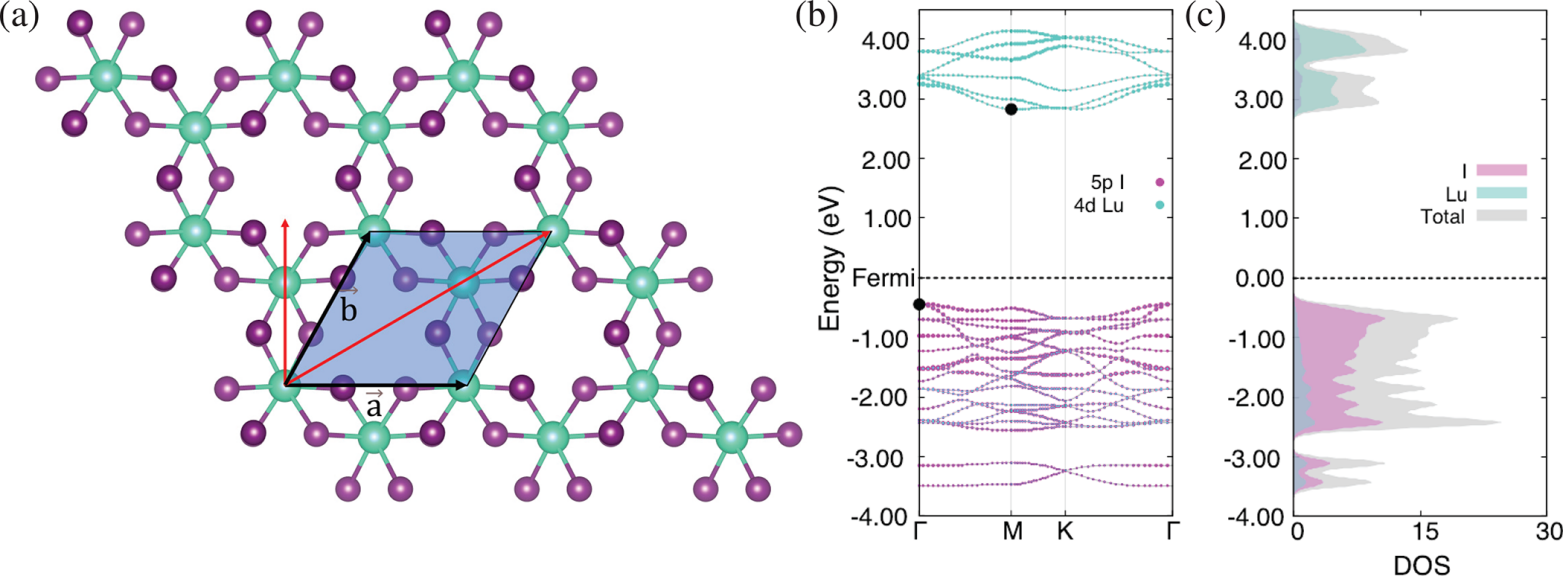
(a) Top view of 1L-LuI_3_. Lu and I atoms are represented by light cyan and dark purple spheres, respectively. The primitive cell, defined by lattice vectors **a** and **b**, corresponds to the shaded area. Armchair NTs are obtained wrapping up the 1L around vector **a**, which in the representation chosen is parallel to the *x*-axis. Zigzag NTs are obtained wrapping up the 1L around the axis, marked in red, parallel to the bisector of the angle defined by the lattice vectors. For simplicity, as discussed in the text, we roll the 1L around the *y*-axis, pointing to a direction that is crystallographically equivalent to the former. (b) Electronic band structure of 1L-LuI_3_; the color code indicates the dominant orbital contribution to each band (Γ = 0.0, 0.0, 0.0; *M* = 0.5, 0.0, 0.0; *K* = 2/3, 1/3, 0.0). (c) Total and projected density of states showing the contributions of atoms belonging to the different atomic species. Color code: cyan = Lu atoms; purple = I atoms.

In this paper we present an exhaustive study of reduced dimensionality nanosystems based on LuI_3_, a prototypical member of the larger series of lanthanide and actinide trihalide family^27^ that also includes LuCl_3_ and LuBr_3_, focusing on 1D nanotubes. LuI_3_ crystallizes with the rhombohedral BiI_3_-type structure^27,28^ containing hexagonal layers with octahedrally coordinated Lu atoms. These layers are built through edge-sharing of the I atoms of one LuI_6_ octahedron with three nearest-neighbor octahedra (see Figure 1a). Because of the occurrence of hollows in such layers, interstitial atoms that can act as dopants are often incorporated into the structure. One of the main applications of LuI_3_ is indeed associated with the ease of doping it with Ce, forming LuI_3_:Ce systems reported to be extremely performing scintillators: a 2% of Ce^3+^ in LuI_3_ yields one of the highest light output for lanthanide trihalide scintillators.^29,30^

We first discuss the electronic structure of the monolayer, bilayer and trilayer and then we move to the layered bulk system, whose crystallographic structure has been recently resolved by synchrotron X-ray diffraction (XRD), and then focus on few-layer 2D systems. We also consider in some detail the structural and electronic properties of LuI_3_ based nanotubes. This is a timely study because it has been recently shown that such nanotubes can be prepared (unpublished results). Then, we study nanotubes of two different chiralities (those corresponding to armchair and zigzag in carbon nanotubes) and four diameters. In particular, we show that for one chirality the bandgap is direct at the Γ point, while in the other is indirect, with the minimum of the conduction band at the zone boundary. Finally, we report a metastable structure, where a diameter-dependent number of I atoms segregate toward the inner wall of the nanotube, giving rise to a one-dimensional metallic state that is protected by the outer insulating nanotubes backbone.

## Computational Methods

We performed density functional theory (DFT) calculations using the Vienna ab initio simulation package (VASP)^31^ and the projector augmented waves (PAW) method.^32,33^ We used an energy cutoff of 176 eV and the generalized gradient approximation (GGA), as parametrized by Perdew, Burke, and Ernzerhof (PBE),^34^ for the exchange-correlation energy. We have performed careful convergence tests on the energy cutoff for the three-layer system, increasing it up to 300 eV. We found that changes of the total energy per unit formula are lower than 0.02%, i.e., 30 meV, while variations of the Kohn–Sham eigenvalues are within 0.09%, i.e., 2.1 meV. The long-range van der Waals interaction is taken into account by the DFT-D3 approach with Becke–Johnson damping.^35,36^ The Brillouin zone was sampled with Monkhorst–Pack^37^ grids of **k**-points for thin layers. We used grids of 12 × 12 × 1, 12 × 12 × 4, and 1 × 1 × 5 for thin layers, bulk, and nanotubes, respectively, taking *xy* to be the plane of the layers and the *z* direction to be parallel to the axis of the nanotubes. Atomic positions were relaxed until all the forces were lower than 0.01 eV/Å, while the in-plane lattice parameter of the single layer was optimized in order to have stresses of at most 3.79 × 10^–4^ GPa. The axial lattice parameter of the nanotubes is then kept fixed to *a* and  for armrchair and zigzag nanotubes, respectively (see discussion below), where *a* is the lattice parameter of the single layer. Projected density of states, orbital-resolved band structures, and wave function analysis were obtained with the VASPKIT postprocessing package.^38^ We are aware that the ideal setup for the calculation of electronic properties of such (and similar) systems^39,40^ should exploit hybrid methods or, alternatively, the GW approach. This is clearly due to the widely reported tendency of DFT to underestimate the experimental bandgap. Nevertheless, the large number of atoms involved in our calculations along with the inclusion of broad portions of vacuum (to avoid spurious interactions among replicas of the tubes) prevented us from using such more accurate (but at the same time computationally expensive) methodologies in computing our systems electronic features. On the other hand, due to the chemical homogeneity of the systems here investigated, we are conscious of the fact that any possible underestimation in the conduction region (the valence is always properly described, being the DFT by definition a ground state theory) should be constant, making the comparison between electronic properties of the investigated systems still extremely reliable and meaningful.

## 2D LuI_3_: From Monolayer to Bulk

### Monolayer

A list of relevant structural parameters for the optimized structure of an ordered LuI_3_ layer (1L-LuI_3_), as in Figure 1, is given in Table 1. 1L-LuI_3_ is a wide-bandgap semiconductor. The bandgap is indirect, as shown in Figure 1b, with the valence band maximum (VBM) at the Γ point and the conduction band minimum (CBM) at the M point. Figure 1c displays the projected density of states (pDOS), where projections on specific atomic orbitals of each element type allow identifying the main character of both valence and conduction band. For valence bands, the electronic structure is dominated by I 5p states, whereas the conduction band is dominated by Lu 4d, with smaller contributions from I 5p levels.

**Table 1:** Lattice Constants, Lu–I Bond Length, and Interlayer Distance of 1L-LuI_3_, 2L-LuI_3_, and 3L-LuI_3_

system	lattice constant (Å)	Lu–I bond length (Å)	interlayer separation (Å)
1L	7.50	2.99	
2L	7.36	2.97	3.40
3L	7.30	2.97	3.41

As it is well-known, semilocal approximations to the exchange-correlation functional, like the one here employed, considerably underestimate the energy bandgap. Indeed, we obtain a bandgap of 3.26 eV, while the experimental value reported for few-layered (i.e., bulk) LuI_3_, a closely related system, is 4.53 eV.^29^ To obtain a quantitatively accurate estimate of the electronic bandgap, we have also performed quasiparticle *single-shot G*_0_*W*_0_ calculations^41,42^ on top of the previously optimized primitive cells. For the quasi-particle calculation of the bandgap we employed a 14 × 14 × 2 Γ-centered mesh of the BZ adding 210 empty states (corresponding to ∼20 eV). 260 frequency grid points were considered in the calculation along with a 140 eV plane-wave cutoff for the response functions. With the mentioned setup, we obtained a value of 5.15 eV.

In order to validate the accuracy of our calculations, we separately considered the role of spin–orbit coupling (SOC), whose effect might be important due to the relatively heavy mass of both Lu and I, and of semicore states in the PAW pseudopotentials. Therefore, with the geometry previously optimized, we recalculated the electronic structure including SOC and adding 4d and 5s semicore states (9 valence electrons vs 25 valence electrons) to the Lu pseudopotential. Differences in the band structure are minimal (see Figure S1). As expected, in the valence band, the degeneracy of the I 5p states is lifted in the low-energy region because of spin–orbit interaction, but neither the band dispersion nor the band character is impacted.

Therefore, we can conclude that our GGA-PBE calculations—without SOC and without Lu semicore states—provide reliable results, and from now on, we are sticking to this computational framework. The only caveat is that the bandgap is underestimated (3.26 vs 5.15 eV obtained with *G*_0_*W*_0_), as usual in this kind of calculation. As a first approximation, nonetheless, we expect the error to be comparable in all the nanostructures considered below.

### Bilayer, Trilayer, and Bulk

**Figure 2:**
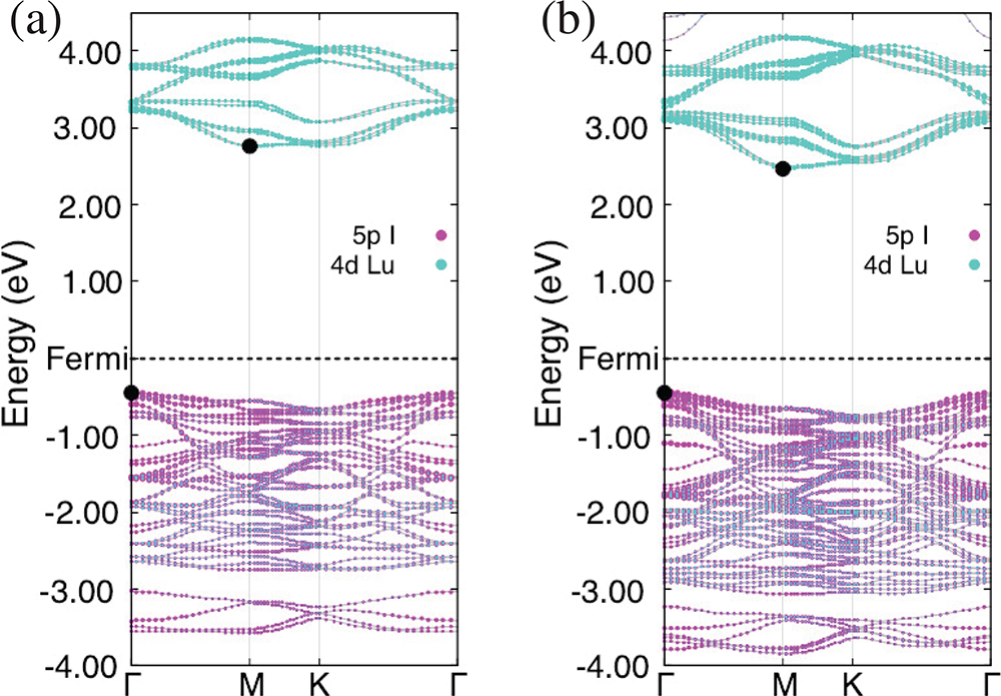
Electronic band structure of (a) 2L- and (b) 3L-LuI_3_.

In LuI_3_ the layers are bound by means of a weak van der Waals interaction, which is reflected by a rather large interlayer separation. We obtain 3.40 and 3.41 Å for the bilayer (2L) and the trilayer (3L), respectively. The effect of such a weak interaction is slightly lifting the degeneracy that one would observe in a system with strictly noninteracting layers. As a result, the electronic structures of 2L and 3L are very similar to each other and with that of 1L, besides some shrinking of the GGA-PBE bandgap that goes from 3.26 to 3.01 to 2.91 eV as the number of layers increases. The band structure diagrams are displayed in Figure 2.

**Figure 3:**
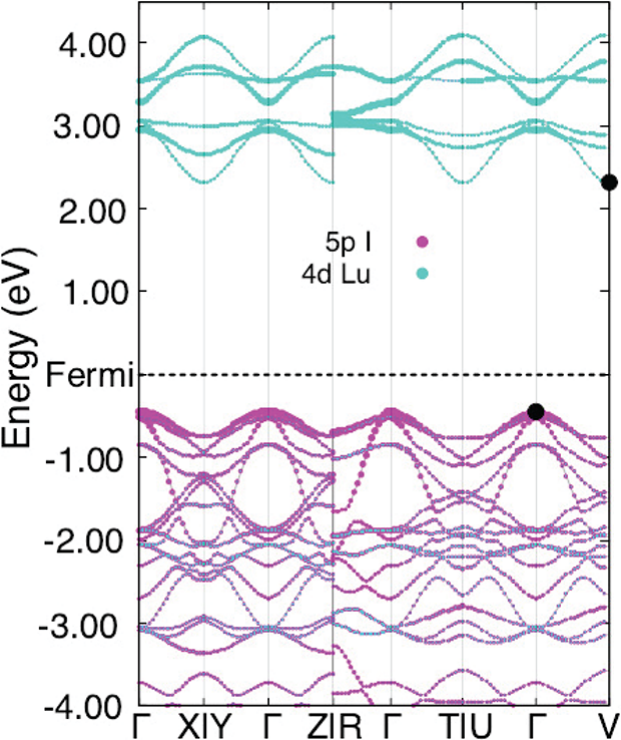
Electronic band structure of rhombohedral *R*3̅ bulk LuI_3_. Γ = 0.0, 0.0, 0.0; X–Y = 0.5, 0.0, 0.0–0.0, 0.5, 0.0; Z–R = 0.0, 0.0, 0.5–0.5, 0.5, 0.5; T–U = 0.0, 0.5, 0.5–0.5, 0.0, 0.5; V = 0.5, 0.5, 0.0.

XRD measurements^29^ showed that in bulk LuI_3_ the sheets arrange in a rhombohedral *R*3̅ (space group 148) crystal structure. However, the more recent refinement (unpublished results) clearly showed that partial occupation of the Lu sites had to be assumed. This implicates that the bulk structure does not contain a perfectly ordered set of identical layers. Parenthetically, it is worth mentioning that in a previous study^43^ the crystal structure was also reported using a hexagonal cell (space group 143), thus pointing out the same conclusion. In order to check if such disorder could have any noticeable influence in our calculations, we performed structural optimizations using either a small perfectly ordered rhombohedral cell or a larger hexagonal cell with more structural freedom. We obtained practically the same energy per unit formula (i.e., a difference of only 8 μeV/f.u.), thus showing that structural disorder does not play any significant role. We found a Lu–I bond length and interlayer separation of 2.98 and 3.32 Å, respectively, to be compared with the values 2.98 and 3.51 Å reported by Zhu and co-workers.^44^ The electronic band structure of the rhombohedral bulk structure (see Figure 3) exhibits an indirect bandgap of 2.76 eV between the VBM at Γ and the CBM at V, consistent with those for the different *n*L-LuI_3_ slabs. For the sake of comparison, in Figure S2 we report the band structure calculated at the same level of theory for the hexagonal bulk cell of LuI_3_.

## Semiconducting LuI_3_ Nanotubes

**Figure 4:**
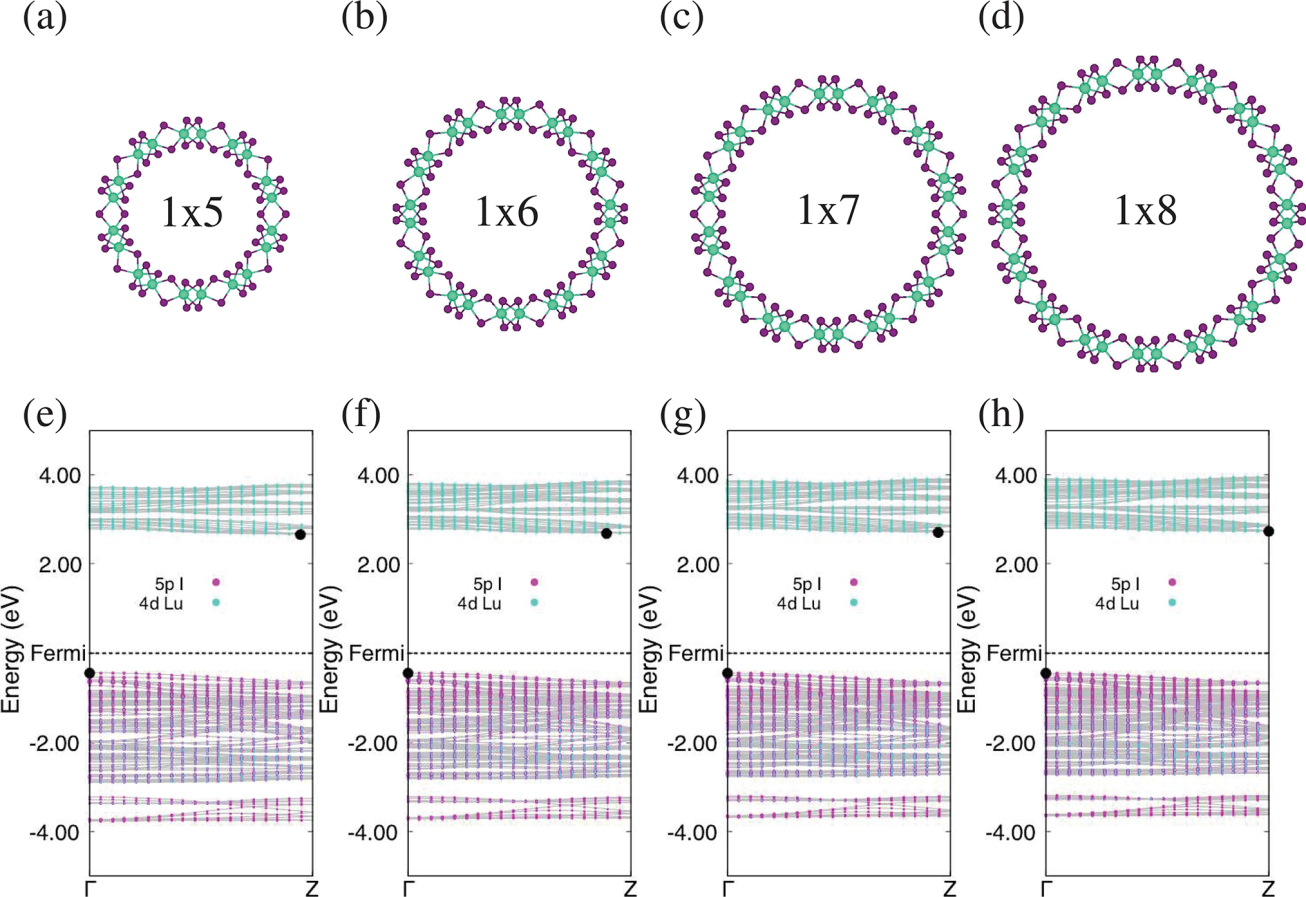
(a–d) Optimized geometry [color code: cyan = Lu atoms; purple = I atoms] and (e–h) electronic band structures of armchair LuI_3_ NTs. Diameters from left to right are 2.2, 2.6, 3.0, and 3.4 nm. The 1 × *N* nomenclature in (a–d) is the same used in Table 2 and refers to the size of supercell of the rectangular unit cell before wrapping.

**Figure 5:**
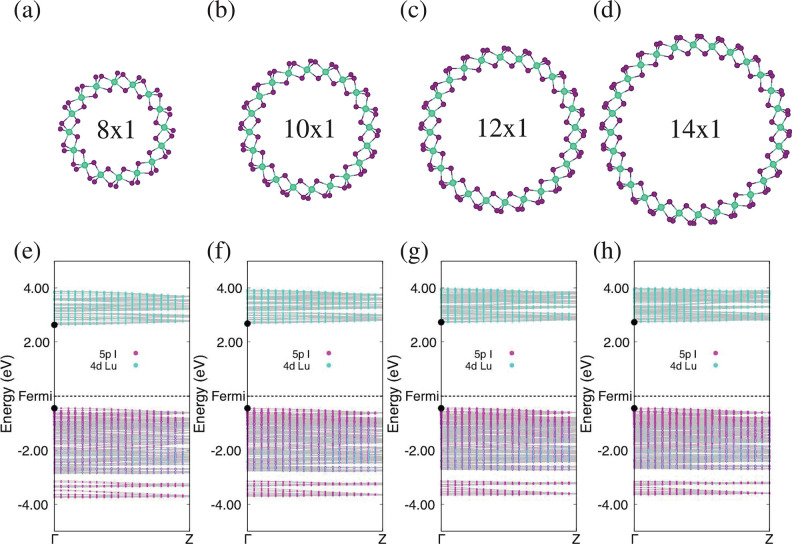
(a–d) Optimized geometry [color code: cyan = Lu atoms; purple = I atoms] and (e–h) electronic band structures of zigzag LuI_3_ NTs. Diameters from left to right are 2.1, 2.5, 3.0, and 3.4 nm. The *M* × 1 nomenclature in (a–d) is the same used in Table 2 and refers to the size of supercell of the rectangular unit cell before wrapping.

We build NTs by wrapping 1L-LuI_3_ around the **a** lattice vector, parallel to the *x*-axis, or around the bisector of the angle defined by the lattice vectors **a** and **b**, as sketched in Figure 1a; see also Table 2. In practice, we considered the rectangular nonprimitive unit cell, constructed 1 × *N* and *M* × 1 supercells, and rolled them up around the *x*-axis and *y*-axis, a direction crystallographically equivalent to the bisector described above. If applied to single-layer graphene, this procedure would give rise to the well-know *armchair* and *zigzag* carbon NTs, and thus we adopt the same nomenclature from now on (indeed, the usual armchair and zigzag profiles can be recognized if displaying only Lu atoms in a side view of the NT). We consider *N* = 5, 6, 7, and 8 and *M* = 8, 10, 12, and 14. This starting geometry is then fully optimized. In this way we obtained NTs of both chiralities that span a diameter range that goes from 2.1 to 3.4 nm, of the order of experimental samples recently prepared (unpublished results). A cross-section view of these armchair and zigzag NTs, after the geometry optimization, is shown in Figures 4 and 5, respectively.

**Table 2:** Selected Parameters of the Semiconducting NTs Investigated^a^

type	SC	*n*	*D*_ext_	*D*_int_	Lu–I	*E*_gap_
arm	1 × 5	80	25.67	18.91	2.978	3.10 (i)
arm	1 × 6	96	29.66	22.80	2.987	3.13 (i)
arm	1 × 7	112	33.79	26.98	2.980	3.16 (i)
arm	1 × 8	128	37.81	30.89	2.988	3.18 (i)
zz	8 × 1	128	24.18	17.05	2.98	3.08 (d)
zz	10 × 1	160	28.80	21.70	2.98	3.15 (d)
zz	12 × 1	192	33.09	26.28	2.98	3.17 (d)
zz	14 × 1	224	30.93	37.98	2.98	3.19 (d)

a Chirality, supercell of the rectangular unit cell before wrapping (SC), number of atoms (*n*), external and internal diameter (*D*_ext_, *D*_int_, Å), Lu–I bond length (Å), and GGA-PBE electronic bandgap (*E*_gap_, eV) [arm: armchair; zz: zigzag. (i): indirect; (d): direct bandgap].

**Figure 6:**
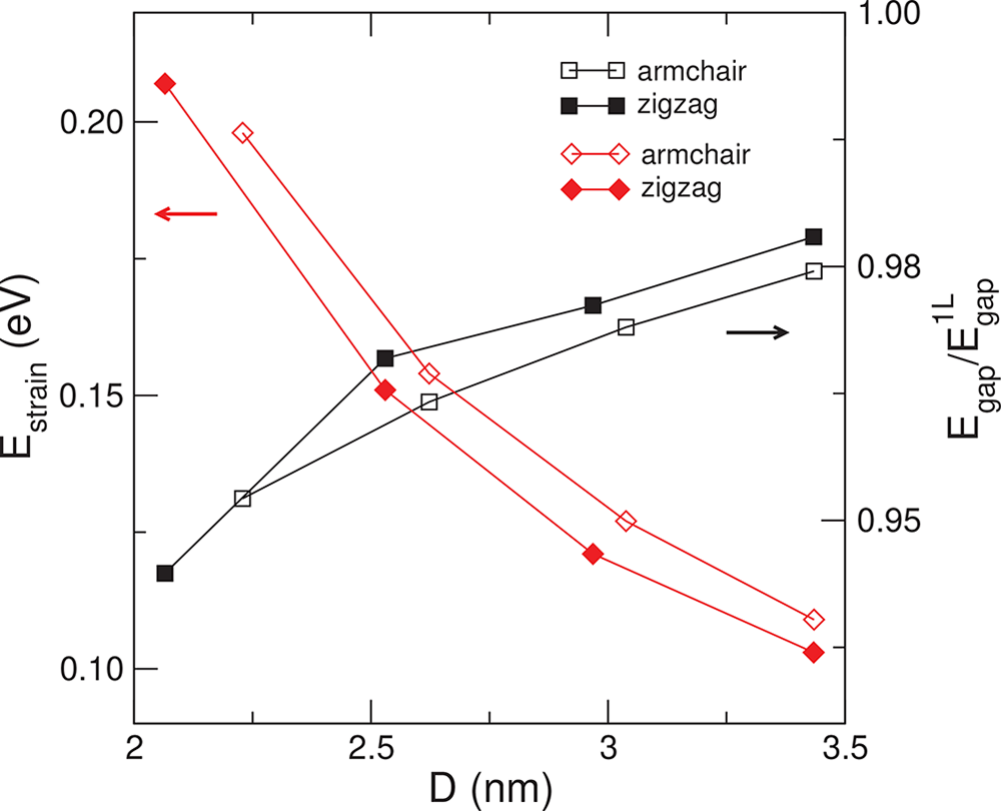
Bandgap and strain energy as a function of the diameter for NTs of both chiralities. The bandgaps of the NTs, *E*_gap_, are given with respect to the bandgap of the 1L, *E*_gap_^1L^.

We obtain bandgaps that are weakly dependent on the NT diameter, similarly to what was previously reported for PbI_2_.^21^ Yet, the trend is for the bandgap to increase with diameter and to tend to the value on 1L-LuI_3_ (equivalent to a NT with infinite diameter and zero curvature). The values are plotted in Figure 6 in units of the bandgap of the 1L, i.e., *E*_gap_/*E*_gap_^1L^, where *E*_gap_ is the bandgap of the nanotube (monolayer). In the same figure we also report the strain energy as a function of the LuI_3_ diameter, which we define as *E*_strain_ = *E*_NT_ – *E*_1L_, where *E*_NT_ and *E*_1L_ are the total energies of the NT and the stand-alone 1L-LuI_3_ per formula unit. As expected, the larger the NT diameter, the lower the energy cost that must be paid to wrap the flat LuI_3_ sheet, as shown in Figure 6. Ideally, indeed, *E*_strain_ = 0 for tubes with zero curvature, i.e., *E*_NT_ = *E*_1L_. Importantly, this plot also indicates that armchair NTs, obtained wrapping 1L-LuI_3_ around the **a**-vector, are slightly favored. From the application point of view, such a large bandgap could be potentially exploited in UV-absorbing systems to protect materials against UV radiation. For the same class of applications, reduced dimensionality oxides of Zn, Ti, and Ce have been similarly reported to perform well.^45^

The character of the bands is similar to the case of the 1L, with valence states dominated by 5p states of iodine atoms and conduction states dominated by 4d states of Lu atoms. What is perhaps the most interesting feature of the electronic structure of these NTs is that their bandgap is *direct* or *indirect*, depending on the chirality. In particular, as shown in Figure 4, armchair NTs have an indirect bandgap, with the VBM at Γ and the CBM next to the zone boundary (roughly speaking, the larger the diameter, the closer to the zone boundary; see the black dots in Figure 4 indicating the band minimum/maximum). Conversely, zigzag NTs have a direct bandgap at Γ, as shown Figure 5. This chirality-dependent behavior is a direct consequence of the band folding of 1L-LuI_3_. When wrapping the monolayer around the *x*-axis, the only periodic direction that survives in reciprocal space is Γ–*M*; the remaining part of the band diagram of Figure 1b is quantized, as a result of the radial confinement and is folded onto the Γ point. As the CBM of the 1L-LuI_3_ was at *M*, it stays there and the bandgap remains indirect. On the other hand, in zigzag NTs the periodic direction is the *y*-axis (or the crystallographically equivalent bisector of the angle defined by the lattice vectors and indicated in Figure 1a), which corresponds to the Γ–*K* path of the Brillouin zone. Therefore, the CBM of the 1L-LuI_3_ is folded onto Γ, and the bandgap becomes direct. This is an important difference because in this case efficient radiative decay can occur without the need of emitting a phonon, as the VBM and the CBM have the same crystalline momentum. Nevertheless, it is worth noting that even for armchair NTs the bandgap is only marginally indirect, with the CBM at Γ only 6–9 meV higher than the absolute CBM at the zone boundary. Notice that due to such a small value, the bandgap is going to be in practice direct at room temperature (and even at temperatures as low as ∼100 K). Finally, it should be reminded that having a direct bandgap is only a necessary condition to observe intense luminescence and that a proper calculation of the intensity of the optical transition, which is not practical due to the large number of atoms involved, should in principle be performed.

## Metallic Nanotubes

**Figure 7:**
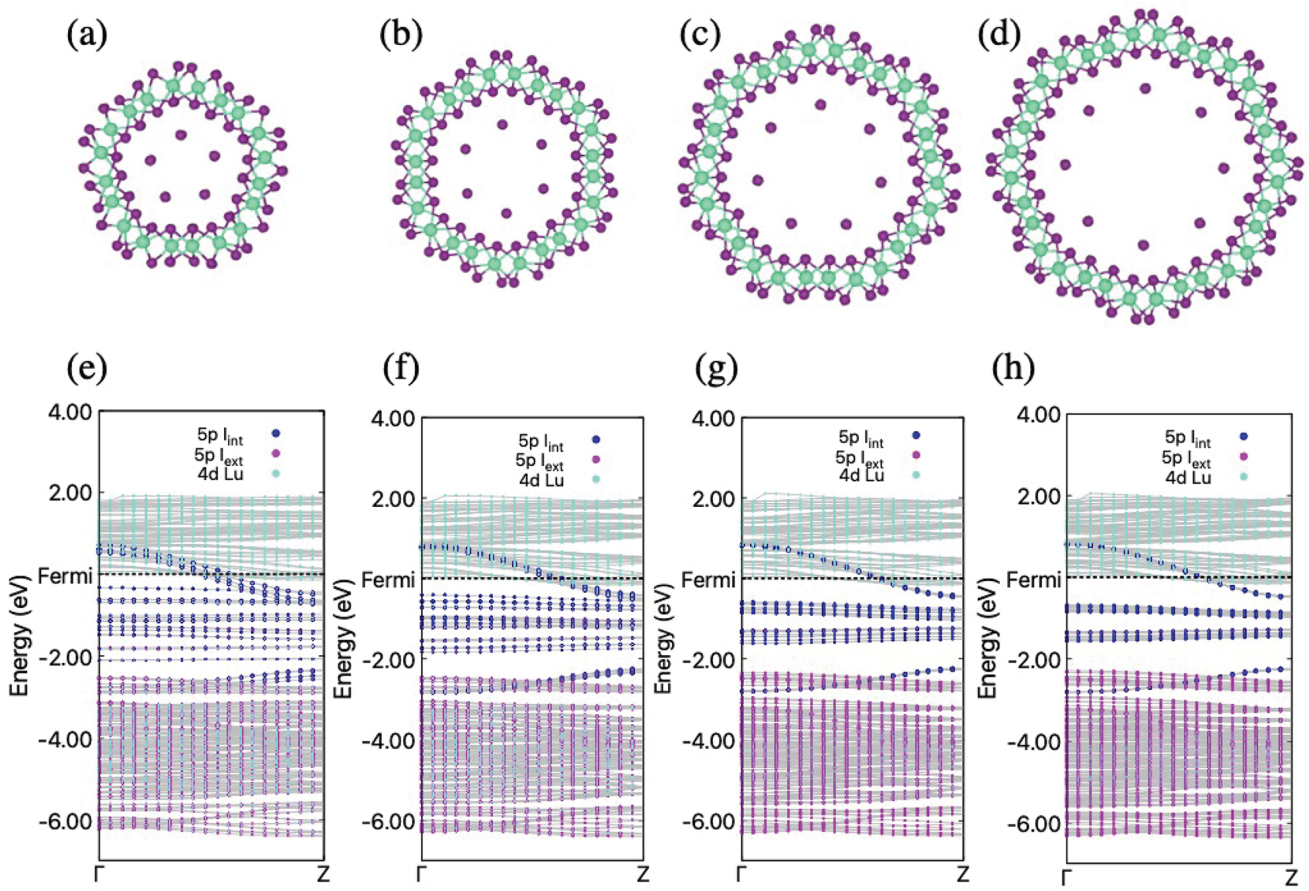
(a–d) Optimized geometry [color code: cyan = Lu atoms; purple = I atoms] and (e–h) electronic band structures of metallic LuI_3_ NTs with *N* = 5–8. Diameters from left to right are 2.0, 2.3, 2.6, and 2.9 nm.

**Figure 8:**
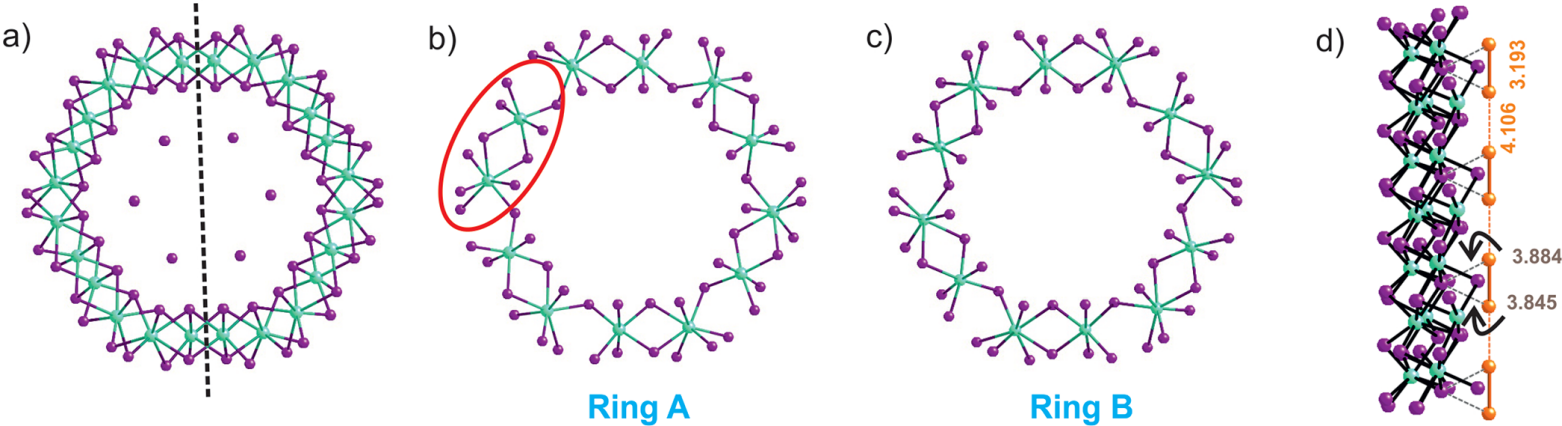
(a) Metastable NT with *N* = 6. (b, c) Rings A and B which build up the backbone (NT′) of the nanotube in (a) by condensation of the iodine atoms.The dashed line in (a) indicates one of the three equivalent glide planes relating two successive rings along the NT′ direction. The elementary building block Lu_2_I_11_ is highlighted in (b). (d) Magnified details of the internal I atoms forming dimers [color code: cyan = Lu atoms; purple = I atoms forming the tube; orange = I atoms forming the dimers].

In this section we report a metastable structure of armchair NTs, which, at the expense of a moderate geometrical rearrangement, results in considerably different electronic properties. In Figure 7 we display NTs of this type with four different diameters, roughly comparable to those of the armchair NTs previously discussed. In these metastable NTs, some I atoms segregate toward the center of the NTs, as clearly visible in Figure 7. These I atoms lie in the hollow part of the NTs at distances ≥3.8 Å from the inner sidewall and are thus bonded to the nanotube backbone via van der Waals interaction (van der Waals radii of I: 2.04 Å). These iodine atoms form noticeably dimerized chains (see Figure 8d). Because there are *N* iodine chains with two atoms as the repeat unit, the general stoichiometric formula of the NT backbone (which from now on we will refer to as NT′) is thus Lu_4*N*_I_12*N*–2*N*_, or simply Lu_2*N*_I_5*N*_ (*N* = 5, 6, 7, 8, ...). Only half of the Lu atoms are hexacoordinated as in all of the systems described up to this point, while the other half are heptacoordinated.

As shown in Figure 8, these NTs can be described as being made of rings containing 2*N* Lu atoms. The elementary unit of one of these rings with general formula Lu_2*N*_I_20*N*_ is a dimeric Lu_2_I_11_ unit (see Figure 8b). These rings build up the NT′ by sharing all the I atoms between two rings, thus leading to NT′ with a Lu_2*N*_I_5*N*_ stoichiometry and Lu_4*N*_I_10*N*_ unit cell, so that two successive rings A and B are related through a glide plane schematically shown as a dashed line in Figure 8. This *packing* within the folded monolayer based on the dimeric Lu_2_I_11_ unit gives rise to the characteristic pentagonal, hexagonal, heptagonal, and octagonal shapes shown in the cross-section views of Figure 7. Note that the I atoms in the hollow part of the NTs form one-dimensional chains that can be seen as resulting from a Peierls distortion, leading to alternating I–I distances along the NT axis of 3.2 and 4.1 Å, i.e., a chain of connected I_2_ molecules. The number of these I_2_ chains of dimers is obviously related to the number of heptacoordinated Lu atoms in a ring, *N*.

The most relevant difference between hexa- and heptacoordinated metal atoms is that the well-known “three-below-two” d-orbital splitting of the octahedral hexacoordinated metal atoms becomes a “two-below-three” d-orbital splitting in heptacoordinated metal atoms.^46^ In other words, the extra ligand simply raises the energy of one of the three almost nonbonding t_2g_ metal orbitals which becomes antibonding. Therefore, as far as electron counting is concerned, the Lu atoms are always in a +3 oxidation state.

These NTs are metallic, as shown in Figure 7. Although the band structure may appear at first sight complex, it is in fact quite simple when the previous structural discussion and the nature of the bands are taken into account. This is more easily seen when looking at the band structure of the *N* = 7 NT (Figure 7g) to which our discussion will be refereed except if otherwise stated. The electronic structure is the superposition of several contributions. First, a large contribution spanning approximately the −6 to −2 eV range which is associated with the occupied states of NT’. The upper bands character is mostly iodine lone pairs. Second, another large contribution starting around the Fermi level up to the highest energies which, except for the lowest part, contains empty antibonding levels of NT′ being mostly Lu d in character. These two contributions are separated by a large gap of 2.66 eV (*N* = 7) which is reminiscent although smaller than that in the semiconducting LuI_3_ nanotubes (3.16 eV for *N* = 7). The reason for the decrease is the raising up of the upper part of the valence band which in all cases tends to separate from the rest of the valence band (see Figure 7). This peak is associated with I lone pairs, and its destabilization simply reflects the more strained nature of the present nanotubes because of the reduced number of I atoms.

Superposed with the NT backbone bands there are four groups of bands (i.e., those in blue in Figure 7), which are those of the inner chains of I dimers. The two sets of flat bands contain 2*N* bands each. The lower set is made of the two π-type bonding orbitals of the iodine dimers which remain as flat bands. This is because the relatively large interdimer contact and the π-type nature of the interdimer interactions result with a very weak interdimer transfer integral along the chain. The upper set is the equivalent for the two antibonding π*-type orbitals of the iodine dimers. It may appear as surprising that the sets of bonding/antibonding π-type bands do not exhibit a larger separation as in the I_2_ molecule. This is due to the relatively large I–I dimer bond length (3.2 Å, see Figure 8d) compared with the usual I–I bonding distance, 2.66 Å. Centered at approximately −3.5 eV and around the Fermi level, there are sets of *N* bands (note that because they overlap with levels of NT′ these bands may not be easily seen for *N* = 5), which are built from the σ and σ* orbitals of the iodine dimers. In contrast with the previous ones, these bands exhibit dispersion because their main component are the I 5p_*z*_ orbitals that point directly along the chain direction so that even if the interdimer separation is long they lead to a substantial interdimer transfer integral. Note that the dispersion of the upper band is noticeably larger. This is due to the fact that the σ* antibonding orbital of the iodine dimer is hybridized toward the outer part of the dimer so as to avoid excessive antibonding interactions, thus increasing the interdimer interaction. In contrast, the σ bonding orbital is hybridized in the opposite direction to maximize the bonding character and the associated transfer integral is weaker.

In principle, the set of *N* upper bands based on the σ* antibonding orbital of the iodine dimer should be empty. It is then surprising that as shown by the band structure of Figure 7, these bands are approximately half-filled, thus conferring metallic character to the system. The reason is that because of the relatively long I–I bond length, the antibonding I–I character is reduced so that these bands are lowered in energy and superpose with the lower part of the NT′ conduction bands. The NT′ unit cell is Lu_4*N*_I_10*N*_ so that because the Lu atoms are in a + 3 oxidation state, there is an excess of 2*N* electrons that should occupy the lower conduction band levels of NT’ if there were not the inner iodine chains. The calculated band structure show that the *N* dispersive bands of the inner I chains are practically half-filled. Consequently, there has been a transfer of approximately *N* electrons from the lower conduction bands of the backbone to the inner chains of I dimers. As a result, the I atoms of these chains are formally I^–0.5^. Note that the lower backbone partially filled conduction bands are considerably narrower that the partially filled bands of the inner I chains so that it is not at all clear that if the latter had not been lowered the nanotube would had been metallic. Most likely, the 2*N* electrons would had been localized, *N* in each ring of NT′. However, the electron transfer ensures that the dispersive inner bands are partially filled and that there are less electrons than Lu centers of the same type (i.e., hexa- or heptacoordinated) in the backbone. Both factors lead to the metallic character of the nanotube.

We must note that the longer than usual I–I bond length is in fact imposed by the backbone. As shown in Figure 8d, each of the two iodine atoms of the dimer makes an I···I contact of 3.8–3.9 Å with one iodine of NT′. These two contacts, which are somewhat shorter than the sum of the van der Waals radii of the two iodines, provide the glue to keep the iodine chains bonded to NT′. Consequently, although the metallicity mostly resides in the inner part of NT, it is in fact imposed by the backbone because, as discussed above, if the I–I distance had been shorter, the σ* bands would be higher in energy, the electron transfer to these bands would not occur, and the electrons would instead reside in the fairly flat bands of NT′. The approximately half-filled nature of the inner chains bands points out the possibility of a Peierls distortion that would stabilize the system. Such dimerization would result with pairs of bonded dimers, i.e., tetrameric iodine units. Because, as discussed above, the iodine spacing is in fact imposed by the NT backbone, such tetramerization would require some reorganization of the backbone so as to induce the new I···I van der Waals contacts appropriate for the tetramerization. Because of the rigidity of the NT backbone, such distortion is probably unlikely. We have computationally tested such a possibility in the hexagonal NT, using a 1 × 1 × 2 supercell that would not forbid the envisaged tetramerization. Yet, we found that I–I distances along the tube axis remain essentially unchanged, thus confirming the stability of the described dimer structure against a further Peierls-like distortion. Finally, let us note that although replacing Lu for other lanthanides would slightly modulate the separation between the two iodine atoms of NT′ implicated in the van der Waals I···I interaction with the dimerized iodine chains, the dimerization imposed by the topology of the backbone is too large to be significantly modified, and a situation with a nondimerized iodine chain is unlikely. Consequently, the electronic structure of these nanotubes should be essentially stable with respect to the nature of the lanthanide.

The previous discussion is applicable to all NTs of this series. The only difference is that when *N* becomes smaller and the NT radius becomes smaller, the inner iodine chains start to interact. As a result, the bands of each of the four groups of bands, but more specially those based on the π and π* orbitals, which being made of I 5p_*x*,*y*_ orbitals interact better, spread into a wider energy range although they keep the same shape. Consequently, the physical behavior should not exhibit any noticeable variation. We note, howeve, that the backbone of the *N* = 5 NT is considerably strained with respect to those with *N* ≥ 6.

Finally, we point out a possible route toward magnetism in these NTs. If one could remove the inner chains of I dimers (they are bonded to the nanotube backbone through I···I contacts only a bit shorter than the sum of the van der Waals radii suggesting, as model calculations also do, that these chains are rather mobile), there would be a retro transfer of *N* electrons to the backbone. In such a way, neutral Lu_4*N*_I_10*N*_ (or simply Lu_2*N*_I_5*N*_) NTs would be obtained where the lower conduction bands would contain 2*N* electrons. As already pointed out, these bands are relatively narrow so that it is most likely that the metallic character would be lost and the electrons would be localized in either the 2*N* hexacoordinated or the 2*N* heptacoordinated Lu atoms. If this prediction is realized, there are several different magnetic configurations that should have similar energies, and this new class of Lu_2*N*_I_5*N*_ NTs could have interesting magnetic properties. For the time being we only tested this possibility by considering a simple spin-polarized ferromagnetic calculation with one unpaired electron in all hexa- or heptacoordinated Lu atoms. Nevertheless, in none of these cases the magnetic ordering turned out to be stable and the charge density readily converged to a nonmagnetic solution. However, as in other magnetic nanotubes^47^ it is likely that more complex magnetic orderings would set in. Further investigations considering different and more complex magnetic orderings, some of which require larger supercells for commensurabilty reasons, are in order before a firm assessment can be given.

A word of caution regarding the predictions presented in this section is in order, as we are aware that we cannot discard any possible dynamical instability of these structures. In order to exclude this possibility, one should compute the phonon spectrum of the NTs and make sure that no imaginary frequencies show up. Unfortunately, the *ab initio* calculation of the phonon dispersion of systems with unit cells of the order of hundreds of atoms is computationally extremely challenging and beyond the scope of this paper.

## Conclusions

The electronic structure of nanostructures based on hexagonal layers of LuI_3_ have been studied by means of first-principles DFT calculations. Both bulk and slabs with different number of layers exhibit large and indirect bandgaps. The latter depend on the number of layers, the variation originating from a widening of the conduction band with the number of layers. A rich variety of nanotubes can be generated from these layers. Semiconducting LuI_3_ nanotubes of two different chiralities have been studied. The direct or indirect nature of the electronic gaps depends on the chirality, and a simple rationalization of this observation is provided based on band folding arguments. Remarkably, a metastable form of the armchair LuI_3_ nanotubes can be obtained under a structural rearrangement such that some iodine atoms are segregated toward the center of the nanotube forming chains of dimerized iodines. These nanotubes with an Lu_2*N*_I_5*N*_ backbone are predicted to be metallic. Both the relatively long intradimer I–I bond length and the excellent overlap between successive dimeric units are crucial for the development of the metallic property. Although the partially filled bands are practically half-filled, the nanotubes are found to be immune toward a Peierls distortion. Because the iodine chains in the inner part of the nanotubes are only weakly bound to the nanotube backbone, it is suggested that it should be possible to remove these chains to generate a new series of neutral Lu_2*N*_I_5*N*_ nanotubes. In view of the electron transfer process associated with the chains removal and the nature of the bands receiving the transferred electrons, it is speculated that the Lu_2*N*_I_5*N*_ nanotubes could exhibit interesting magnetic properties. The proposed new metastable nanotubes are thus potentially very challenging so that we urge experimental work to test our suggestions. In addition, we note that the LuI_3_ structure is exhibited by a large number of lanthanide and actinide trihalides so that a subtle tuning of the electronic, optical, transport, and probably magnetic properties of these nanotubes may be attained.
