# Supplementary material for: Chirality Effects and Semiconductor versus Metallic Nature in Halide Nanotubes
Source: J Phys Chem C Nanomater Interfaces. 2023 Apr 5;127(15):7162–71. doi: 10.1021/acs.jpcc.3c00244 (PMC10124746; doi:10.1021/acs.jpcc.3c00244)
Supplement: Supplementary file 1 — jp3c00244_si_001.pdf [file jp3c00244_si_001.pdf]

Supporting Information: Chirality Effects and Semiconductor vs. Metallic Nature in Halide Nanotubes

Costanza Borghesi,[†] Giacomo Tanzi Marlotti,[‡,¶] Enric Canadell,[*,¶] Giacomo Giorgi,[*,†,§,‖] and Riccardo Rurali[*,¶]

†*Department of Civil & Environmental Engineering (DICA), Università degli Studi di Perugia, Via G. Duranti 93, 06125 Perugia, Italy*

‡*Department of Physics "Aldo Pontremoli", Università degli Studi di Milano, Via Celoria 16, I-20133 Milano, Italy*

¶*Institut de Ciència de Materials de Barcelona, ICMAB–CSIC, Campus UAB, 08193 Bellaterra, Spain*

§*CIRIAF - Interuniversity Research Centre, University of Perugia, Via G. Duranti 93, 06125 Perugia, Italy*

‖*CNR-SCITEC, 06123 Perugia, Italy*

E-mail: canadell@icmab.es; giacomo.giorgi@unipg.it; rrurali@icmab.es

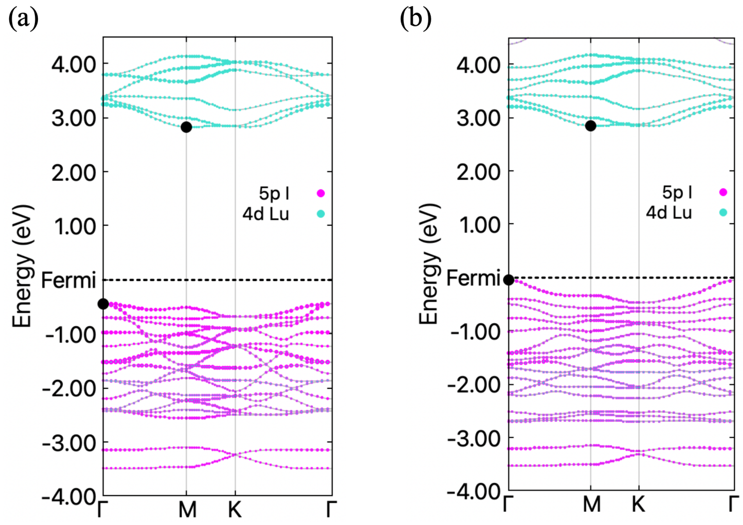

Figure S1: Band structure calculations for 1L-LuI_3 (a) without and (b) with the inclusion of relativistic effects (SOC).

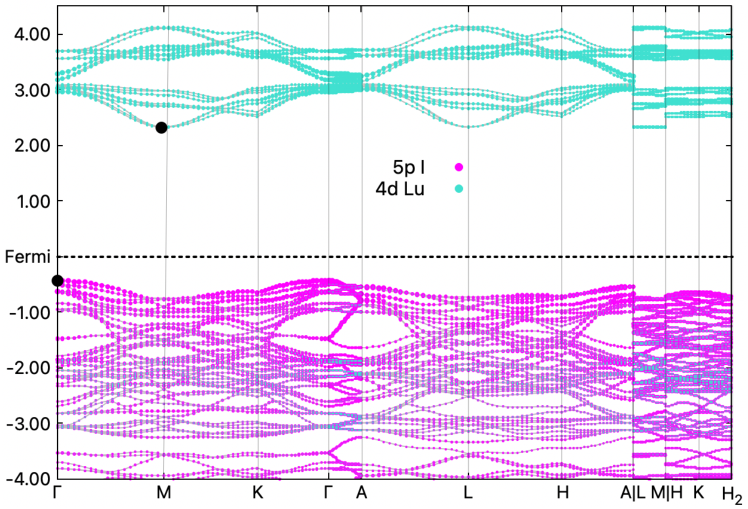

Figure S2: Electronic band structure of hexagonal bulk LuI_3. [Γ=0.0, 0.0, 0.0; M= 0.5, 0.0, 0.0; K= 0.333, 0.333, 0.0; A= 0.0, 0.0, 0.5; L=0.5, 0.0, 0.5; H= 0.333, 0.333, 0.5; $H_2 = 0.333, 0.333, -0.5$]
